# In-Depth Exploration of Chemical Constituents from *Justicia procumbens* L. Through UHPLC-Q-Exactive Orbitrap Mass Spectrometry

**DOI:** 10.3390/molecules30173554

**Published:** 2025-08-30

**Authors:** Liangjun Guan, Huibin Luo, Siqiong Liu, Xinrong Ming, Mengdie Hu, Lan Luo, Jingyi Tan, Shunli Xiao

**Affiliations:** School of Pharmaceutical Sciences, Hunan University of Medicine, Huaihua 418000, China; guanlj3@163.com (L.G.); lhb202405@163.com (H.L.); liusq649@163.com (S.L.); mingxr132418@163.com (X.M.); 13982454687@163.com (M.H.); 15073612512@163.com (L.L.); tanjyyyyyy@163.com (J.T.)

**Keywords:** *Justicia procumbens* L., phytochemical compounds, identification, UHPLC-Q-Exactive Orbitrap MS

## Abstract

*Justicia procumbens* L. (JP) has been traditionally used to treat colds with fever, swollen and sore throat, jaundice, malaria and eczema. Studies indicate that lignans constitute the primary bioactive components, yet systematic phytochemical investigations remain limited. Therefore, it is necessary to establish a rapid and effective method to identify the chemical components in JP. In this study, ultra-high-performance liquid chromatography-quadrupole-Exactive Orbitrap mass spectrometry (UHPLC-Q-Exactive Orbitrap MS) coupled with parallel reaction monitoring (PRM) was used for the first time to investigate JP. Based on chromatographic retention times, MS and MS² data, and bibliography data, a total of 132 compounds were tentatively identified, including 54 lignans, 19 flavonoids, 31 organic acids, 18 alkaloids, and 10 other types of constituents. Among these, 77 compounds are reported for the first time in JP, including 14 potential novel compounds. These results provide valuable reference and data support for the study of pharmacodynamic substances and quality control of this medicinal plant.

## 1. Introduction

*Justicia procumbens* L. (JP), derived from the dried whole plant of the Acanthaceae family, is one of the most important herbs used in different countries [1,2]. It is primarily used to treat colds with fever, swollen and sore throat, jaundice, malaria and eczema [3]. JP exhibits diverse pharmacological activities, including anti-inflammatory, anti-asthma, antiviral and antiplatelet aggregation activity [4,5,6,7]. Current research on JP constituents primarily relies on traditional separation and purification methods for compound identification [8,9,10]. These approaches, however, are operationally cumbersome and time-consuming. To date, the major identified compounds include lignans, alkaloids, and triterpenoids, while characterization of other components remains limited [3]. Consequently, the chemical composition of JP is inadequately studied. There is therefore a need to establish a comprehensive methodology for rapid analysis and identification of its constituents. This would enhance understanding of its material basis and improve quality control measures.

Liquid chromatography-mass spectrometry (LC-MS) is a cornerstone analytical technique renowned for its high accuracy and rapid analysis of complex samples [11,12]. Its success is fundamentally driven by instrumental advancements [13], particularly in mass analyzer technology. Critical systems for pharmaceutical analysis include time-of-flight (TOF), ion traps (ITs), quadrupoles (Q), and Orbitrap instruments, delivering high resolution, sensitivity, and mass accuracy across wide dynamic ranges [14]. Prominent hybrid configurations, such as quadrupole time-of-flight (Q-TOF), triple quadrupole (QQQ), quadrupole-Orbitrap (Q-Orbitrap), and ion trap-Orbitrap (IT-Orbitrap), further enhance these capabilities [15,16]. For instance, UHPLC-Q-Exactive Orbitrap MS enables rapid and accurate analysis of chemical components in plant samples, offering advantages such as high precision of analytical results and low detection limits [17,18]. While the commonly employed Full MS/data-dependent MS² (Full MS/dd-MS²) mode acquires sample data, this approach presents challenges for analyzing minor components [19]. Recent studies demonstrate that compared to the full scan mode, the Parallel Reaction Monitoring (PRM) mode significantly enhances detection sensitivity [20]. This study consequently endeavors to establish a novel analytical method integrating UHPLC-Q-Exactive Orbitrap MS with PRM for systematic characterization of phytoconstituents in JP.

In this study, the chemical constituents of JP were rapidly detected and characterized using UHPLC-Q-Exactive Orbitrap MS. This is the first systematic investigation of the chemical composition of JP, and the results will contribute to clarifying the bioactive phytoconstituents of this plant and lay the foundation for the quality control of drugs based on JP in future clinical applications.

## 2. Results and Discussion

### 2.1. Analytical Strategy

To achieve a comprehensive characterization of the chemical constituents in JP and enhance the accuracy of compound identification, an effective analytical strategy was developed. First, an in-house database of chemical constituents in JP was constructed with the information of trivial name, molecular formula, and chemical structure of each compound by searching multiple available databases (SciFinder, Web of Science, and CNKI, etc.). Second, the MS spectra of ten reference standards, including lignans, flavonoids, and organic acids, were collected and their fragmentation pattern were analyzed. Third, a highly effective analytical method employing UHPLC-Q-Exactive Orbitrap MS coupled with PRM was used for the acquisition of the MS^2^ data. Based on the abovementioned identification strategy, the chemical components of JP were identified through comparison with standards, diagnostic fragment ions, as well as by referencing the literature and an in-house library.

### 2.2. Characterization of the Chemical Composition in JP by LC-MS/MS

**Figure 1:**
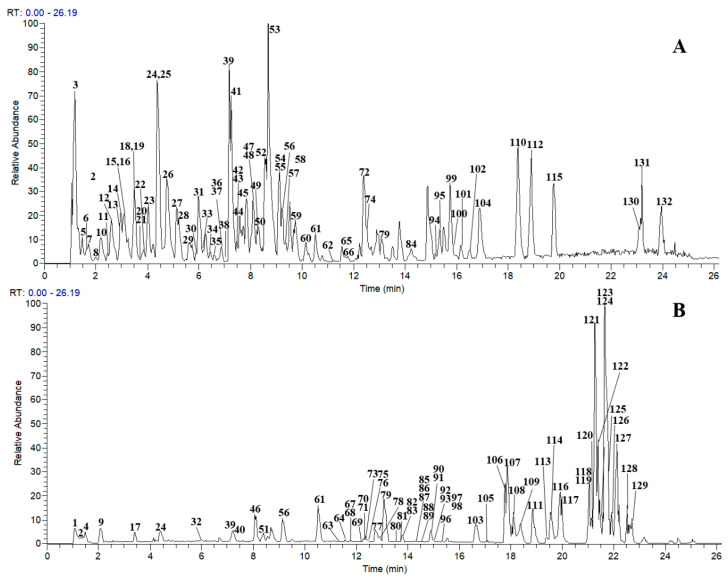
The base peak chromatograms of JP obtained by UHPLC-Q-Exactive Orbitrap MS. (**A**) negative ion mode. (**B**) positive ion mode.

A total of 132 compounds were tentatively identified in JP, including 54 lignans, 19 flavonoids, 31 organic acids, 18 alkaloids, and 10 other types of constituents. Among these, 10 compounds were definitively identified using reference standards. The chromatographic and mass data of those detected constituents are summarized in Table S1, and the corresponding base peak ion (BPI) profiles are presented in Figure 1.

#### 2.2.1. Identification of the Lignans in JP

Lignans are a class of polyphenols that are widely found in edible and terrestrial plants. These compounds, along with their glycosides, are the main phytoconstituents of JP. Numerous types of lignans have been reported; they can be categorized into two subtypes, arylnaphthalene- and dibenzylbutane-based, based on their skeletons. Arylnaphthalene lignans have the phenyl-naphthyl skeleton, which is the most abundant type of component in JP. Dibenzylbutane lignans are molecules with two benzene rings in their structure [21]. A total of 54 lignans were characterized in the JP extract.

**Figure 2:**
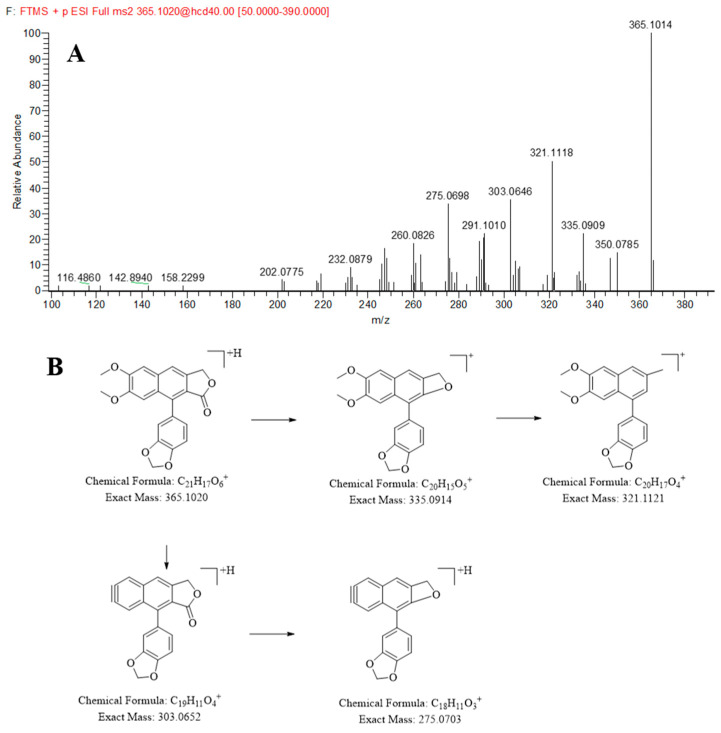
The MS/MS spectrum (**A**) and proposed fragmentation patterns (**B**) of Justicidin B.

Compounds **117** and **121** were observed at 20.04 and 21.27 min and identified as justicidin B and justicidin A, respectively, by comparing the retention time and MS data with reference standards. Taking justicidin B as an example, the protonated ion [M + H]^+^ at *m/z* 365.1007 was observed in positive ion mode. In the MS/MS spectrum of justicidin B, *m/z* 335.0909 was generated by losing CO from *m/z* 365.1007, and then the loss of an oxygen atom gave *m/z* 321.1118. *m/z* 303.0646 was produced by losing two methoxy groups from [M + H]^+^, followed by loss of CO to give a fragment ion at *m/z* 275.0698. Its MS/MS spectrum and possible cleavage pathways are shown in Figure 2.

Compound **62** was eluted at 11.28 min, possessing the quasi-molecular ion [M-H]^-^ at *m/z* 531.1518, a fragment ion at *m/z* 351.0877 was obtained by the sequential loss of a hexose (162 Da) and H_2_O (18 Da) from *m/z* 531.1518, then the loss of a methyl group gave *m/z* 336.0642. Therefore, it was identified as the procumbenoside K [9]. Compounds **63** and **69** were eluted at 11.30 and 12.18, respectively, and possessed the same [M + H]^+^ at *m/z* 529.1343. The main product ions at *m/z* 367.0805 were attributed to the loss of a hexose (162 Da). Therefore, they were identified as being procumbenoside I or its isomer [9].

Compound **64** had a quasi-molecular ion [M + H]^+^ at *m/z* 559.1450, *m/z* 397.0912 [M + H-162]^+^, and 323.0906 [M + H-162-C_2_H_2_O_3_]^+^ were observed in the MS^2^ spectrum; it was deduced as justatropmer I [22]. Compounds **70** and **75** had the same molecular weight and yielded consistent fragment ions at *m/z* 397.0912 as **64**; therefore, they were characterized as justatropmer E and justatropmer F. Likewise, compound **76** was identified as justatropmer C or justatropmer D. Compound **87** was tentatively characterized as cilinaphthalide A-glu, which is a new compound worthy of further separation and identification.

Compound **78** produced an adduct ion at *m/z* 600.3015 [M + NH_4_]^+^ with a molecular formula of C_29_H_42_O_12_. In the position ion mode, the precursor ion [M + NH_4_]^+^ yielded two characteristic fragment ions at *m/z* 151.0752 and 181.0856, which belong to the characteristic ions of dibenzylbutane lignan. Therefore, it was tentatively identified as 5-methoxy-4,4’-di-O-methylsecolarciresinol diacetate+C_2_H_6_O_3_. Likewise, Compounds **109**, **114**, **119**, **123**, **124**, **125**, and **128** obtained the pseudo molecular ions at *m/z* 463.2326 (C_25_H_34_O_8_), 508.2543 (C_26_H_34_O_9_), 492.2225 (C_25_H_30_O_9_), 522.2695 (C_27_H_36_O_9_), 475.2324 (C_26_H_34_O_8_), 492.2589 (C_26_H_34_O_8_), and 489.2116 (C_26_H_32_O_9_), respectively. They were deemed to be the 5-methoxy-4,4’-di-O-methylsecolarciresinol, justin C, justin B, 5-methoxy-4,4’-di-O-methylsecolarciresinol diacetate, 5-methoxy-4,4’-di-O- methylsecolarciresinol, secoisolariciresinol dimethyl ether diacetate, (-)-dihydroclusin diacetate by matching with the literature [21,23].

Compounds **81** and **89** were eluted at 13.67 and 14.62 min, yielding quasi-molecular ion [M + H]^+^ at *m/z* 675.1923. Fragment ion *m/z* 381.0961 [M + H-162-132]^+^ was observed, indicating the presence of one glucose and one apiose, and *m/z* 363.0855, 333.0751 and 305.0803 were generated. Therefore, they were identified as procumbenoside B or its isomer. Compounds **83**, **88**, **92**, **96**, **93**, and **98** yielded quasi-molecular ion [M + H]^+^ at *m/z* 543.1498, 543.1498, 645.1816, 645.1816, 777.2236, and 571.1606, respectively, which had the same fragment ion at *m/z* 381.0961. Thus, they were assigned as justicidinoside C/procumbenoside D/cleistanthin B, procumbenoside A/procumbenoside H, procumbenoside E /cliatoside A/aizin, and jsticidinoside A.

Compounds **90**, **97**, **106**, and **107** possessed the same quasi-molecular ion [M + H]^+^ at *m/z* 411.1074 and characteristic fragment ions at *m/z* 137.0231, they were characterized as isomer of isomer of 6’-hydroxy justicidin A. Compounds **112** and **116** also gave same quasi-molecular ion [M + H]^+^ at *m/z* 411.1074, among them, compound **112** was deduced as 6’-hydroxy justicidin A according to the peak intensity [24] and **116** was preliminary identified as cilinaphthalide B [25]. Compound **91** produced an adduct ion at *m/z* 590.1870 [M+NH_4_]^+^ with a molecular formula of C_28_H_28_O_13_. It produced two daughter ions at *m/z* 411.1066 and 137.0232 and was identified as justicidinoside A by searching the literature [26].

Compound **94** was observed at 15.18 min, processing the quasi-molecular ion [M-H]^-^ at *m/z* 907.2528. The fragment ions at *m/z* 379.0824, 319.0612 were generated by losing the sugar chain and C_2_H_4_O_2_, and it was identified as being ciliatoside B. The molecular weight of compound **100** was 12 Da more than of **94**, it showed same fragment ion with those of **94**, and tentatively identified as cliatoside B + C. Compounds **95**, **101**, **110**, and **115** sowed the pseudo molecular ions at *m/z* 395.0773 (C_21_H_16_O_8_), 409.0931 (C_22_H_18_O_8_), 379.0824 (C_21_H_16_O_7_), 363.0513 (C_20_H_12_O_7_), respectively. They were tentatively identified as 9-Hydroxy-5-(4-hydroxy-3,5-dimethoxyphenyl)-furo [3′,4′:6,7] -naphtho[2,3-d][1,3]dioxol-6(8H)-one, 6’-hydroxy justicidin C, 6’-hydroxy justicidin B, and taiwanin E by searching the in-house database.

Compounds **118**, **122** and **126** exhibited the quasi-molecular ion at *m/z* 395.1125, along with fragment ion patterns comparable to those of justicidin A. Therefore, they were identified as the isomer of justicidin A. By searching the in-house library, the abovementioned compounds might be the following structures: justicidin C, phyllamyricin C, chinensinaphthol methyl ether, 5’-Methoxy retrochinensin, or procumphthalide A.

#### 2.2.2. Identification of the Flavonoids in JP

Flavonoids, commonly found in various plants, are a class of polyphenolic compounds having a basic structural unit of 2-phenylchromone. In plants, flavonoids are involved in many biological processes and in response to various environmental stresses [27]. Flavonoid compounds have attracted much attention due to their wide biological applications. Over six thousand distinct flavonoids have been characterized and cataloged. These bioactive compounds are significant elements in human nutrition, offering a wide array of health advantages. These include immunomodulatory, anti-inflammatory, antibacterial, antiviral, antineoplastic, anti-allergic, anti-mutagenic, vasodilatory and cardioprotective effects [28]. UHPLC-Q-Exactive Orbitrap MS analysis tentatively identified 19 individual flavonoid constituents in the JP samples.

**Figure 3:**
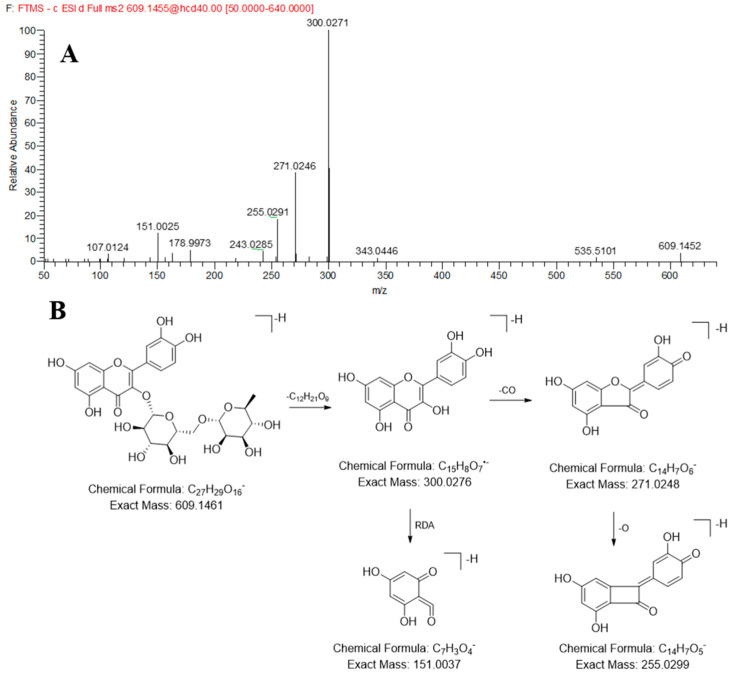
The MS/MS spectrum (**A**) and proposed fragmentation patterns (**B**) of Rutin.

Compounds **38**, **45**, **47**, **50** and **65** exhibited pseudomolecular ions [M − H]^−^ at *m/z* 447.0940 (C_21_H_20_O_11_), 609.1472 (C_27_H_30_O_16_), 463.0890 (C_21_H_20_O_12_), 463.0889 (C_21_H_20_O_12_), 445.0782 (C_21_H_18_O_11_), respectively, were unambiguously identified as orientin, rutin, hyperoside, isoquercitrin, and baicalin by comparing their accurate mass information and chromatography retention times with reference standards. Taking rutin as an example, the quasi-molecular ion [M − H]^−^ at *m/z* 609.1472 was observed in negative ion mode. In the MS/MS spectrum of rutin, the main fragment ion at *m/z* 300.0271 was generated by losing a glucose and rhamnose from *m/z* 609.1472, then the neutral loss of the CO and CO_2_ gave *m/z* 271.0271 and 255.0271, *m/z* 151.0025 was generated by Diels-Alder (RDA) rearrangement. Its MS/MS spectrum and possible cleavage pathways are shown in Figure 3.

Compounds **39**, **41** and **44** were eluted at 7.17, 7.29 and 7.57 min and yielded the same quasi-molecular ion [M − H]^−^ at *m/z* 509.1314; the fragment ions were observed at *m/z* 300.0277, 271.0249, 255.0298, which were similar to those of rutin. Considering the difference of 14 Da in molecular weight compared to rutin, they were tentatively characterized as quercetin 3-O-sambubioside or its isomer by comparing the MS/MS spectrum of the mzVault database. Compound **40** produced a quasi-molecular ion [M + H]^+^ at *m/z* 465.1029 with a molecular formula of C_21_H_20_O_12_, which is the same as isoquercitrin. Therefore, it was identified as an isomer of isoquercitrin.

Compounds **43**, **52**, **53**, **54**, and **56** possessed the same quasi-molecular ion at *m/z* 609. 1450, which is the same as that of rutin. However, the main fragment ion of **43** was *m/z* 284.0327, it was tentatively identified as kaempferol 3-O-gentiobioside by searching the literature [29]. The fragment ion profiles of compounds **52–54** and **56** exhibited a high degree of similarity, and a signal at *m/z* 315.0514 was detected in MS^2^ spectra; thus, they were characterized as nelumboroside A or its isomer.

Compound **49** was eluted at 8.25 min with deprotonated ion [M − H]^−^ at *m/z* 579.1364; the fragment ions at *m/z* 284.0327, 255.0298, 227.0347 were detected, and it was tentatively identified as leucoside by searching the literature [30]. Similarly, compounds **55** and **57** had the same molecular weight but different intensity fragment ions, and were assigned as isorhamnetin-3-O-nehesperidine and narcissoside through comparison of their MS^2^ spectra with the database. Compounds **58** and **59** yielded deprotonated ions [M − H]^−^ at *m/z* 477.1044 and were tentatively identified as cacticin or its isomer.

#### 2.2.3. Identification of the Organic Acids in JP

**Figure 4:**
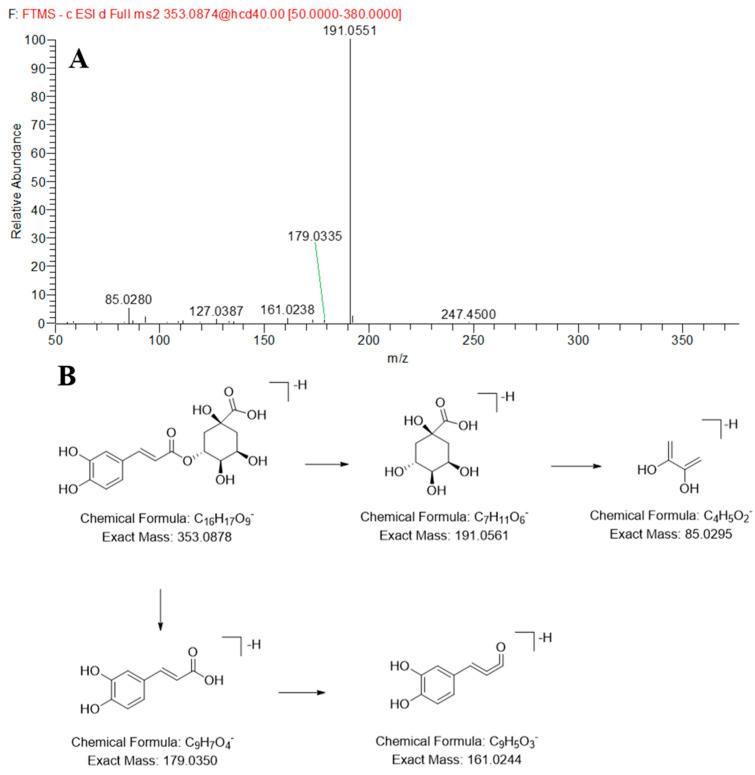
The MS/MS spectrum (**A**) and proposed fragmentation patterns (**B**) of Chlorogenic acid.

Compounds **15**, **24**, and **60** were observed at 3.08, 4.38, and 10.15 min and were accurately characterized as neochlorogenic acid, chlorogenic acid and azelaic acid, respectively, by comparing the retention time and MS data with those of reference standards. Using chlorogenic acid as an example, it produced a base peak ion at *m/z* 191.0551 in negative ion mode, which belongs to the signal of quinic acid, then further fragmentation led to the formation of a fragment ion at *m/z* 85.0280. The fragment ion at *m/z* 179.0335 is attributed to caffeic acid, which further generated a fragment ion at *m/z* 161.0238. Its MS/MS spectrum and possible cleavage pathways are shown in Figure 4. Compound **29** showed the same molecular weight as the chlorogenic acid and was inferred as an isomer of chlorogenic acid.

Compounds **6**, **10**, **12** and **14** with the same deprotonated ion [M − H]^−^ at *m/z* 371.0623 (C_15_H_16_O_11_) were eluted at 1.57, 2.19, 2.61 and 2.97. The fragment ions at *m/z* 209.0297, 191.0190, and 85.0281 were detected in the MS^2^ spectrum of those compounds, indicating they belong to the phenolic acid class of components. They were tentatively identified as 2-O-caffeoylglucaric acid or its isomer by referring to the literature data [31]. Likewise, Compounds **7** and **13** produced the same deprotonated ion [M − H]^−^ at *m/z* 369.0466 (C_15_H_14_O_11_) and were characterized as 2-O-caffeoylglucarate or its isomer.

Compounds **16**, **21–23**, **25–27** were eluted at 3.08, 3.70, 3.85, 4.04, 4.38, 4.74, and 5.14 min and showed the same deprotonated molecular ion [M − H]^−^ at *m/z* 385.0782 (C_16_H_18_O_11_). Compared with compound **6**, they have an additional methylene group. Thus, they were tentatively inferred as 2-O-feruloyl glucaric acid or its isomer. Compounds **18**, **19**, **35**, **36**, **99**, **102**, and **104** were eluted at 3.48, 3.48, 6.57, 6.86, 15.74, 16.53, and 16.89 min, with the deprotonated ions [M − H]^−^ at *m/z* 285.0618 (C_12_H_14_O_8_), 315.0727 (C_13_H_16_O_9_), 367.1039 (C_17_H_20_O_9_), 225.1131 (C_12_H_18_O_4_), 227.1286 (C_12_H_20_O_4_), and 329.2335 (C_18_H_34_O_5_), respectively. They were assigned as 2,3-dihydroxybenzoic acid 3-O-*β*-D-xyloside, 5-(*β*-D-Glucopyranosyloxy)-2-hydroxybenzoic acid, 4-O-feruloyl-quinic acid or its isomer, corchorifatty acid F, 3Z-dodecenedioic acid and tianshic acid according to the annotations in the database.

#### 2.2.4. Identification of the Alkaloids in JP

Cyclopeptide alkaloids with a 14-membered ring, justicianene A, were first discovered by Jin et al. in JP in 2015 [8]. Cyclopeptide alkaloids are defined as polyamidic basic compounds found in many higher families of plants, and are usually composed of a tyrosine-derived 4 (or 3)-hydroxystyrylamine moiety, a common amino acid as a ring-bonded amino acid residue, and a *β*-hydroxy amino acid unit connected to the styryl fragment via an ether bridge [32]. Up to now, four cyclopeptide alkaloids have been isolated and identified in JP. A total of 18 alkaloids were characterized in JP, among which 11 were classified as cyclopeptide alkaloids, including seven potential novel compounds.

Compounds **61**, **67**, and **120** were found at 10.52, 11.78 and 21.16 and yielded quasi-molecular ions [M + H]^+^ at *m/z* 547.3493, 581.3336 and 570.2598. Compared with the literature information, they were inferred to be justicianene D, justicianene C and justicianene A, respectively. Compounds **68**, **71**, **73**, **77**, and **79** showed the quasi-molecular ions [M + H]^+^ at *m/z* 615.31793 (C_35_H_42_N_4_O_6_), 595.3492 (C_33_H_46_N_4_O_6_), 629.3336 (C_36_H_44_N_4_O_6_), 615.3177 (C_35_H_42_N_4_O_6_), and 629.3332 (C_36_H_44_N_4_O_6_), of which compounds **68** and **77** showed the same fragment ion at *m/z* 114. 1278 as justicianene C. The remaining compounds had a main fragment ion at *m/z* 120.0808, which was attributed to 2-phenylethan-1-imine, a partial structure of justicianene C. Therefore, they were tentatively identified as dehydro-justicianene C + C_3_, justicianene C + CH_2_, justicianene C + C_4_, dehydro-justicianene C + C_3_, and justicianene C + C_4_, respectively. Compound **48** exhibited the identical fragment ions at *m/z* 180.0657, 155.0815, 293.1507, 129.1051, and 249.1606 in the MS/MS spectra of positive mode of as justicianene D, and was therefore characterized as deethyl-justicianene D based on the predicted molecular formula.

Compounds **1**, **4**, **9**, and **51** produced quasi-molecular ions [M + H]^+^ at *m/z* 118.0865 (C_5_H_11_NO_2_), 221.0921 (C_11_H_12_N_2_O_3_), 166.0862 (C_9_H_11_NO_2_), and 537.3284 (C_27_H_44_N_4_O_7_), respectively, and were identified as betaine, farylhydrazone C, 1-carboxy-2-phenylethanaminium, and glidobactin G based on database annotations. Compound **46** showed the same fragment ions as glidobactin G and was tentatively inferred to be dehydrated-glidobactin G.

#### 2.2.5. Other Chemical Constituents in JP

By comparing the precise molecular weight and MS/MS information with the literature data, 10 other compounds were preliminarily identified. Compound **84** had an adduct ion peak [M+NH_4_]^+^ at *m/z* 505.2660, and the molecular formula was assigned to C_23_H_40_O_9_ by limiting the measurement error to 5 ppm. The *m/z* 59.0125 was assigned to a fragment of acetic acid, while *m/z* 417.2487 was inferred by losing C_2_H_3_O from [M+NH_4_]^+^. According to the reference information, it was inferred to be cosmosporaside B [33].

Compounds **28**, **30**, and **33** were detected at 5.25, 5.90, and 6.17 min, respectively, and possessed the same adduct ion peak [M+NH_4_]^+^ at *m/z* 449.2034. The characteristic daughter ion at *m/z* 59.0125 was observed in MS/MS spectra, indicating that they had a fragment of acetic acid. Based on the predicted molecular formula of C_19_H_32_O_9_, they were tentatively identified as cosmosporaside B-C_4_H_8_. Similarly, compounds **8** and **72** were deduced as hydroxy-cosmosporaside B-C_4_H_6_. Compounds **34** and **72** exhibited the same molecular weight and were tentatively characterized as an isomer of forsythoside E. Compounds **17** and **20** were identified as tryptophan and an isomer of aucubin, respectively.

Chinese herbal medicine resources are the material basis for the prevention of disease in Traditional Chinese Medicine (TCM). Natural products derived from them are an important source of lead compounds and have made significant contributions to the discovery of new drugs [34]. Each herb contains hundreds of bioactive constituents and exhibits a diverse structural profile. The conventional methodology for identifying bioactive constituents, which primarily relies on separation and purification techniques, is both time-consuming and labor-intensive and involves extensive repetitive procedures. MS is the most selective technique for the rapid qualitative determination of known compounds as well as the identification of unknown compounds from the extracts of natural products. JP, recorded in many herbal works, has a complex chemical composition and contains lignans, flavonoids, alkaloids, terpenoids, and other active components. Modern pharmacological investigations indicated that lignans are the main chemical components in JP, which exhibit various pharmacological activities, including antitumor, antivirus, and inhibition of platelet aggregation. In our study, several novel lignan constituents were identified through the application of MS, enabling targeted isolation and assessment of their biological activities. The newly identified cyclopeptide alkaloids also represent a promising source of lead compounds [35].

## 3. Materials and Methods

### 3.1. Chemicals, Reference Standard and Materials

Chromatographic-grade methanol was obtained from Merck (Branchburg, NJ, USA). LC–MS-grade formic acid was purchased from Thermo Fisher Scientific (Carlsbad, CA, USA), and HPLC-grade water was sourced from Watson Water (Guangzhou, China). All other solvents were of analytical grade. Details of the 10 reference standards are provided in Table S2. JP(20241105) procured from Lianqiao Herbal Medicine Market (Shaoyang, China) was authenticated as JP by Professor Cai Wei (Hunan University of Medicine).

### 3.2. Standard and Sample Preparation

A 3.00 g aliquot of dried JP powder was accurately weighed and extracted with 25 mL of methanol via sonication for 1 h. The extract was centrifuged (12,000 rpm, 10 °C, 10 min), and the supernatant was filtered and subjected to LC-MS analysis.

The 10 reference standards were accurately weighed and dissolved in methanol to prepare stock solutions (1 mg/mL). These were serially diluted to working concentrations, stored at 4 °C, and brought to room temperature prior to analysis.

### 3.3. Instruments and LC–MS/MS Conditions

Qualitative analyses were carried out using a Q-Exactive Focus Orbitrap Mass Spectrometer (Thermo Fisher Scientific, Carlsbad, CA, USA). A GN-A nitrogen generator (Greenville Scientific LLC, China) supplied nitrogen for ionization. Separations used a Waters BEH C18 column (100 × 2.1 mm, 1.7 μm) at 40 °C. The mobile phase consisted of (A) 0.1% formic acid and (B) acetonitrile at a flow rate of 0.3 mL/min. The following gradient program was applied: 0–12 min, 5–18%B; 12–19 min, 18–45%B; 19–21 min, 45–60%B; 21–25 min, 60–80%B; 25–26 min, 80–5%B; 26–30 min, 5–5%B.

MS analysis was performed in both positive and negative ionization modes using electrospray ionization (ESI) in the scan range of *m/z* 100–1500, and two separate acquisitions were made for each polarity. The additional conditions for MS analysis were as follows: sheath gas, 30; auxiliary gas, 10; spray voltage, 3.0 kV for negative ESI and 3.5 kV for positive ESI; capillary temperature, 320 °C; and auxiliary gas heater temperature, 350 °C. The MS^1^ spectra were acquired in full MS mode at a resolution of 35,000, whereas MS^2^ spectra were obtained by ddMS^2^ or PRM mode triggered by inclusion ions at a resolution of 17,500. The normalized collision energy (NEC) was set as 30%, and the automatic gain control (AGC) target was set to 5.0 × 10^5^.

### 3.4. Data Processing and Analysis

All high-resolution MS data (full-scan MS and MS²) were acquired using Xcalibur software v4.2 (Thermo Fisher Scientific, San Jose, CA, USA). Peaks with intensities exceeding 100,000 counts were selected for identification. Elemental compositions of precursor and fragment ions from selected peaks were calculated using the integrated formula predictor with mass accuracy ≤ 10 ppm. The prediction parameters were constrained as: C [0–90], H [0–90], O [0–50], N [0–10].

All high-resolution MS data were acquired and processed using Xcalibur™ software (v4.1) integrated with Compound Discoverer™ 3.0 software (Thermo Fisher Scientific, San Jose, CA, USA). Data were analyzed through the TCM workflow template, where compounds were identified by spectral matching against reference libraries: mzCloud™ and the Orbitrap Traditional Chinese Medicine Library (OTCML). Peaks with intensities exceeding 100,000 counts were selected for identification. Elemental compositions of precursor and fragment ions from selected peaks were calculated using the integrated formula predictor with mass accuracy ≤ 10 ppm. The prediction parameters were constrained as: C [0–90], H [0–90], O [0–50], N [0–10].

## 4. Conclusions

This research developed an effective strategy that tentatively identified 132 compounds in JP by using UHPLC Q-Exactive Orbitrap MS in full scan mode coupled with PRM, including 54 lignans, 19 flavonoids, 31 organic acids, 18 alkaloids, and 10 other types of constituents. Notably, 77 of these compounds were reported for the first time in JP, substantially expanding the known chemical profile of this ethnomedicinally important plant. JP holds considerable value in traditional medicine systems, particularly for treating conditions like fever, respiratory infections (sore throat), and inflammatory skin disorders (eczema), underpinned by documented anti-inflammatory, antiviral, and antiplatelet activities. However, the chemical basis for these pharmacological effects has remained largely unexplored and poorly defined. This significant gap has hindered the standardization of JP materials, the optimization of extraction processes, and the rigorous validation of its traditional uses through modern pharmacological paradigms. The tentative identification of these 132 constituents, especially the 77 that are identified for the first time in JP, contributes to the essential chemical foundation to fill this gap.

## Data Availability

The original contributions presented in this study are included in the article/Supplementary Materials. Further inquiries can be directed to the corresponding authors.

## Supplementary Materials

The following supporting information can be downloaded at: https://www.mdpi.com/article/10.3390/molecules30173554/s1, Table S1: The detailed information of identified components in JP; Table S2: Detailed information of the 10 reference standards.
