# Supplementary material for: In-Depth Exploration of Chemical Constituents from Justicia procumbens L. Through UHPLC-Q-Exactive Orbitrap Mass Spectrometry
Source: Molecules. 2025 Aug 30;30(17):3554. doi: 10.3390/molecules30173554 (PMC12430035; doi:10.3390/molecules30173554)
Supplement: Supplementary file 1 [file molecules-30-03554-s001.zip › molecules-3824471-supplementary.pdf]

Table S1. The detailed information of identified components in JP

No	t_R/min	Experimental Mass m/z	Theoretical Mass m/z	Error (ppm)	Formula	MS/MS fragment (-)	MS/MS fragment (+)	Identification
1	1.08	118.0865	118.0863	2.12	$C_5H_{11}NO_2$		MS^2[118]:59.0736(15), 72.0813(9)	Betaine
2	1.17	268.1041	268.1040	0.11	$C_{10}H_{13}N_5O_4$		MS^2[268]:136.0616(100), 150.0341(17)	Adenosine
3	1.18	191.0191	191.0186	2.25	$C_6H_8O_7$	MS^2[191]:111.0076(100), 87.0074(46), 85.0282(30), 129.0183(6)		Citric acid
4	1.47	221.0921	221.0921	0.32	$C_{11}H_{12}N_2O_3$		MS^2[221]:162.0547(100), 204.0653(53), 134.0599(40)	Farylhydrazone C
5	1.49	219.0772	219.0764	3.70	$C_{11}H_{12}N_2O_3$	MS^2[219]:144.0444(100), 132.0443(76), 74.0233(42), 158.0601(17)		5-Hydroxytryptophan
6	1.57	371.0623	371.0609	3.83	$C_{15}H_{16}O_{11}$	MS^2[371]:85.0281(100), 209.0297(71), 191.0190(34)		2-O-caffeoylglucaric acid or its isomer
7	1.71	369.0466	369.0452	3.71	$C_{15}H_{14}O_{11}$	MS^2[369]:85.0281(100), 191.0190(10)		2-O-caffeoylglucarate or its isomer
8[#]	1.99	467.2138	467.2123	3.23	$C_{19}H_{34}O_{10}$	MS^2[467]:71.0125(100), 101.0231(88), 161.0445(74),289.1658(67), 113.0232(54)		Hydroxy-Cosmosporaside B-C_4H_6
9	2.09	166.0862	166.0863	-0.06	$C_9H_{11}NO_2$		MS^2[166]:120.0809(100)	1-Carboxy-2-phenylethanaminium
10	2.19	371.0622	371.0609	3.56	$C_{15}H_{16}O_{11}$	MS^2[371]:85.0281(100), 209.0297(71), 191.0190(34)		2-O-caffeoylglucaric acid or its isomer
11	2.44	218.1032	218.1023	4.17	$C_9H_{17}NO_5$	MS^2[218]:88.0390(100), 146.0811(52), 71.0488(16)		Pantothenic acid
12	2.61	371.0621	371.0609	3.29	$C_{15}H_{16}O_{11}$	MS^2[371]:85.0281(100), 209.0297(84), 191.0190(42)		2-O-caffeoylglucaric acid or its isomer
13	2.89	369.0466	369.0452	3.79	$C_{15}H_{14}O_{11}$	MS^2[369]:85.0281(100), 191.0190(10)		2-O-caffeoylglucarate or its isomer
14	2.97	371.0623	371.0609	3.83	$C_{15}H_{16}O_{11}$	MS^2[371]:85.0281(100),		2-O-caffeoylglucaric acid or its

						209.0297(86), 191.0190(42)		isomer
15*	3.08	353.0883	353.0867	4.53	$C_{16}H_{18}O_9$	MS^2[353]:191.0555(100), 135.0441(57),179.0342(55)		Neochlorogenic acid
16	3.08	385.0781	385.0765	4.08	$C_{16}H_{18}O_{11}$	MS^2[385]:85.0281(100), 209.0295(7), 191.0189(11)		2-O-feruloyl glucaric acid or its isomer
17	3.41	205.0972	205.0972	0.24	$C_{11}H_{12}N_2O_2$		MS^2[205]:146.0598(100),188.0702(46), 118.0651(32)	Tryptophan
18	3.48	285.0618	285.0605	4.70	$C_{12}H_{14}O_8$	MS^2[285]:108.0204(100), 152.0104(91)		2,3-dihydroxybenzoic acid 3-O-β-D-xyloside
19	3.48	315.0727	315.0711	5.24	$C_{13}H_{16}O_9$	MS^2[315]:109.0282(100), 153.0183(67)		5-(β-D-Glucopyranosyloxy)-2-hydroxybenzoic acid
20	3.70	345.1195	345.1180	4.38	$C_{15}H_{22}O_9$	MS^2[345]:89.0231(100), 59.0125(98), 71.0125(55)		isomer of Aucubin
21	3.70	385.0782	385.0765	4.34	$C_{16}H_{18}O_{11}$	MS2[385]:85.0281(100), 209.0295(7), 191.0189(11)		2-O-feruloyl glucaric acid or its isomer
22	3.85	385.0781	385.0765	4.08	$C_{16}H_{18}O_{11}$	MS^2[385]:85.0281(100), 209.0295(7), 191.0189(11)		2-O-feruloyl glucaric acid or its isomer
23	4.04	385.0780	385.0765	3.82	$C_{16}H_{18}O_{11}$	MS^2[385]:85.0281(100), 209.0295(7), 191.0189(11)		2-O-feruloyl glucaric acid or its isomer
24*	4.38	353.0881	353.0867	3.97	$C_{16}H_{18}O_9$	MS^2[353]: 191.0555(100), 161.0238(2), 179.0342(1),85.0280 (6)	MS^2[355]:163.0387(100), 145.0282(21)	Chlorogenic acid
25	4.38	385.0781	385.0765	4.08	$C_{16}H_{18}O_{11}$	MS2[385]:85.0281(100), 209.0295(7), 191.0189(11)		2-O-feruloyl glucaric acid or its isomer
26	4.74	385.0780	385.0765	3.82	$C_{16}H_{18}O_{11}$	MS^2[385]:85.0281(100), 209.0295(7), 191.0189(11)		2-O-feruloyl glucaric acid or its isomer
27	5.14	385.0782	385.0765	4.34	$C_{16}H_{18}O_{11}$	MS^2[385]:85.0281(100), 209.0295(7), 191.0189(11)		2-O-feruloyl glucaric acid or its isomer
28#	5.25	449.2034	449.2017	3.72	$C_{19}H_{32}O_9$	MS^2[449]:89.0230(100), 119.0337(36), 59.0124(88)		Cosmosporaside B - C_4H_8
29	5.76	353.0882	353.0867	4.33	$C_{16}H_{18}O_9$	MS^2[353]:191.0555(100)		isomer of Chlorogenic acid
30#	5.90	449.2035	449.2017	3.94	$C_{19}H_{32}O_9$	MS^2[449]:89.0230(100), 119.0337(36), 59.0124(88)		Cosmosporaside B - C_4H_8
31	5.98	387.1664	387.1650	3.75	$C_{18}H_{28}O_9$	MS^2[387]:59.0125(100), 207.1020(18), 163.1118(9)		{(1R,2R)-2-[(2Z)-5-(Hexopyranosyloxy)-2-penten-1-yl]-3-oxocyclopentyl}acetic acid
32	6.00	406.2072	406.2072	0.22	$C_{18}H_{28}O_9$		MS^2[406]:85.0653(100),	Tuberonic acid glucoside

							131.0855(86), 149.0959(81)	
33#	6.17	449.2034	449.2017	3.72	$C_{19}H_{32}O_9$	MS^2[449]:89.0230(100), 119.0337(36), 59.0124(88)		Cosmosporaside B - C_4H_8
34	6.46	461.1673	461.1654	4.23	$C_{20}H_{30}O_{12}$	MS^2[461]:101.0231(59), 269.1030(88), 161.0446(72)		isomer of Forsythoside E
35	6.57	367.1039	367.1024	4.22	$C_{17}H_{20}O_9$	MS^2[367]:191.0554(59),93.0 333(49), 173.0448(32)		4-O-feruloyl-quinic acid or its isomer
36	6.86	225.1131	225.1121	4.31	$C_{12}H_{18}O_4$	MS^2[225]:59.0125(100), 97.0646(3)		Tuberonic acid
37	6.86	387.1666	387.1650	4.26	$C_{18}H_{28}O_9$	MS^2[387]:59.0125(100), 207.1020(18), 163.1118(9)		{(1R,2R)-2-[(2Z)-5-(Hexopyran osyloxy)-2-penten-1-yl]-3-oxoc yclopentyl}acetic acid
38*	6.94	447.0940	447.0922	4.07	$C_{21}H_{20}O_{11}$	MS^2[447]:327.0509(59), 357.0616(36), 299.0560(18)		Orientin
39	7.17	595.1314	595.1294	3.34	$C_{26}H_{28}O_{16}$	MS^2[595]:300.0277(100), 271.0249(28), 255.0298(15)	MS^2[597]:303.0494(100)	Quercetin 3-O-sambubioside or its isomer
40	7.19	465.1029	465.1028	0.34	$C_{21}H_{20}O_{12}$		MS^2[465]:303.0493(100)	isomer of Isoquercitrin
41	7.29	595.1315	595.1294	3.55	$C_{26}H_{28}O_{16}$	MS^2[595]:300.0277(100), 271.0249(28), 255.0298(15)		Quercetin 3-O-sambubioside or its isomer
42	7.46	461.1671	461.1654	3.79	$C_{20}H_{30}O_{12}$	MS^2[461]:89.0230(100), 149.0444(34), 59.0124(52)		isomer of Forsythoside E
43	7.46	609.1472	609.1450	3.63	$C_{27}H_{30}O_{16}$	MS^2[609]:284.0327(100), 255.0299(43), 227.0347(19)		Kaempferol 3-O-gentiobioside
44	7.57	595.1315	595.1294	3.65	$C_{26}H_{28}O_{16}$	MS^2[595]:300.0277(100), 271.0249(28), 255.0298(15)	MS^2[597]:303.0494(100)	Quercetin 3-O-sambubioside or its isomer
45*	7.82	609.1473	609.1450	3.73	$C_{27}H_{30}O_{16}$	MS^2[609]: 300.0277(100), 271.0252(36), 255.0299(20),151.0025(12)		Rutin
46#	8.09	519.3178	519.3177	0.19	$C_{27}H_{42}N_4O_6$		MS^2[519]:100.1123(100)	Dehydrated-Glidobactin G
47*	8.11	463.0890	463.0871	4.10	$C_{21}H_{20}O_{12}$	MS^2[463]:300.0274(100), 271.0247(36), 255.0297(16), 151.0026(10)		Hyperoside
48#	8.11	517.3038	517.3021	3.31	$C_{27}H_{42}O_6N_4$	MS^2[517]:180.0657(100), 155.0815(90), 293.1507(52), 129.1051(58), 249.1606(35)		deethylate-Justicianene D
49	8.25	579.1364	579.1344	3.38	$C_{26}H_{28}O_{15}$	MS^2[579]:284.0327(100), 255.0298(41), 227.0347(20)		Leucoside

50*	8.33	463.0889	463.0871	3.89	$C_{21}H_{20}O_{12}$	MS^2[463]:300.0276(100), 271.0249(40), 255.0299(19), 151.0026(10)	MS^2[465]:303.0493(100)	Isoquercitrin
51	8.38	537.3284	537.3283	0.24	$C_{27}H_{44}N_4O_7$		MS^2[537]:100.1123(100)	Glidobactin G
52	8.58	609.1469	609.1450	3.12	$C_{27}H_{30}O_{16}$	MS^2[609]:314.0434(100), 299.0199(41), 271.0250(29), 243.0297(26)	MS^2[611]:317.0651(100), 302.0415(19)	Nelumboroside A or its isomer
53	8.70	609.1468	609.1450	2.94	$C_{27}H_{30}O_{16}$	MS^2[609]:299.0199(100), 315.0513(63), 271.0250(49)		Nelumboroside A or its isomer
54	9.12	609.1470	609.1450	3.27	$C_{27}H_{30}O_{16}$	MS^2[609]:300.0277(100), 315.0514(82), 271.0250(41)		Nelumboroside A or its isomer
55	9.12	623.1628	623.1607	3.40	$C_{28}H_{32}O_{16}$	MS^2[623]:314.0433(100), 299.0197(79), 271.0249(40), 243.0296(24)		Isorhamnetin-3-O-nehesperidine
56	9.23	609.1473	609.1450	3.76	$C_{27}H_{30}O_{16}$	MS^2[609]:300.0277(100), 315.0514(82), 271.0250(41)		Nelumboroside A or its isomer
57	9.37	623.1627	623.1607	3.31	$C_{28}H_{32}O_{16}$	MS^2[623]:315.0511(100), 300.0275(54), 271.0248(29), 243.0295(16)	MS^2[625]:317.0651(100), 302.0415(14)	Narcissoside
58	9.52	477.1044	477.1028	3.46	$C_{22}H_{22}O_{12}$	MS^2[477]:314.0432(100), 243.0295(58), 271.0246(53), 285.0404(36), 257.0453(20)	MS^2[479]:317.0652(100), 302.0417(23)	Cacticin or its isomer
59	9.77	477.1044	477.1028	3.46	$C_{22}H_{22}O_{12}$	MS^2[477]:314.0432(100), 243.0295(52), 271.0246(51), 285.0404(32), 257.0453(13)		Cacticin or its isomer
60*	10.15	187.0970	187.0965	2.78	$C_9H_{16}O_4$	MS^2[187]:125.0960(100), 97.0646(12)		Azelaic acid
61	10.52	547.3493	547.3493	0.00	$C_{29}H_{46}N_4O_6$	MS^2[545]:180.0657(100), 155.0815(56), 293.1508(62), 129.1021(53), 249.1605(39)	MS^2[547]:114.1278(100)	Justicianene D
62	11.28	531.1518	531.1497	3.93	$C_{26}H_{28}O_{12}$	MS^2[531]:351.0877(65), 336.0642(18)		Procumbenoside K
63	11.30	529.1345	529.1341	0.77	$C_{26}H_{24}O_{12}$		MS^2[529]:367.0805(100),335.0543(74), 317.0437(58), 349.06697(45)	Procumbenoside I or its isomer
64	11.56	559.1450	559.1446	0.73	$C_{27}H_{26}O_{13}$		MS^2[559]:397.0912(76), 295.0959(57), 323.0906(53)	Justatropmer I

65*	11.68	445.0783	445.0765	3.86	$C_{21}H_{18}O_{11}$	MS^2[445]:269.0455(100), 241.0506(3)		Baicalin
66	11.77	312.1246	312.1230	5.03	$C_{18}H_{19}NO_4$	MS^2[312]:148.0519(100), 178.0500(58), 135.0440(25), 190.0501(21), 297.1005(24)		Prototenellin B
67	11.78	581.3336	581.3334	0.45	$C_{32}H_{44}N_4O_6$		MS^2[581]:114.1278(100)	Justicianene C
68#	11.78	615.3179	615.3177	0.36	$C_{35}H_{42}N_4O_6$		MS^2[615]:114.1278(100)	dehydro-Justicianene C+C_3
69	12.18	529.1343	529.1341	0.55	$C_{26}H_{24}O_{12}$		MS^2[529]:367.0805(100)	Procumbenoside I or its isomer
70	12.21	793.2187	793.2186	0.16	$C_{36}H_{40}O_{20}$		MS^2[793]:397.0911(59), 231.0648(44), 295.0959(20), 259.0598(26)	Justatropmer E/Justatropmer F
71#	12.30	595.3492	595.3490	0.39	$C_{33}H_{46}N_4O_6$		MS^2[595]:120.0807(100)	Justicianene C+CH_2
72	12.40	467.2137	467.2123	2.89	$C_{19}H_{34}O_{10}$	MS^2[467]:71.0125(100), 101.0231(88), 161.0445(74),289.1658(67), 113.0232(54)		Hydroxy-Cosmosporaside B-C_4H_6
73#	12.41	629.3336	629.3334	0.38	$C_{36}H_{44}N_4O_6$		MS^2[629]:120.0808(100)	Justicianene C+ C_4
74	12.51	923.2479	923.2452	2.96	$C_{41}H_{48}O_{24}$	MS^2[923]:395.0773(100), 351.0876(93),322.0849(33), 307.0615(28)		Justatropmer G/Justatropmer H
75	12.52	793.2188	793.2186	0.24	$C_{36}H_{40}O_{20}$		MS^2[793]:397.0908(66),231. 0649(33), 295.0959(19), 259.0595(21)	Justatropmer E/Justatropmer F
76	12.52	661.1765	661.1763	0.30	$C_{31}H_{32}O_{16}$		MS^2[661]:397.0908(37), 295.0959(21), 259.0595(18)	Justatropmer C/Justatropmer D
77#	12.75	615.3178	615.3177	0.07	$C_{35}H_{42}N_4O_6$		MS^2[615]:114.1278(100)	dehydro-Justicianene C+C_3
78#	12.92	600.3015	600.3015	0.10	$C_{29}H_{42}O_{12}$		MS^2[600]:151.0752(100), 181.0856(57)	5-Methoxy-4,4'-di-O-methylsecolarciresinol diacetate+$C_2H_6O_3$
79#	13.11	627.3198	627.3177	3.33	$C_{36}H_{44}N_4O_6$	MS^2[627]:180.0658(100), 343.1818(1), 257.1294(45), 387.1721(21),	MS^2[629]:120.0808(100)	Justicianene C+ C_4
80	13.52	349.0707	349.0707	-0.03	$C_{20}H_{12}O_6$		MS^2[349]:275.0697(100), 247.0749(55), 319.0594(52), 217.0644(52)	Justicidin E
81	13.67	675.1924	675.1920	0.62	$C_{32}H_{34}O_{16}$		MS^2[675]:381.0961(100), 363.0855(38), 333.0751(32), 305.0803(32)	Procumbenoside B or its isomer

82	13.78	381.0967	381.0969	-0.42	$C_{21}H_{16}O_7$		MS[2][381]:307.0957(100), 337.1064(83), 279.1010(69)	Neojusticin C/Chinensinaphthol/4'-Demethylchinensinaphthol/Diphyllin/Isodiphyllin
83	13.78	543.1498	543.1497	0.26	$C_{27}H_{26}O_{12}$		MS[2][543]:381.0965(100), 363.0859(73), 332.0678(68)	Justicidinoside C/Procumbenoside D/Cleistanthin B
84	14.26	505.2660	505.2643	3.29	$C_{23}H_{40}O_9$	MS[2][505]:59.0125(100), 417.2487(6)		Cosmosporaside B
85	14.33	739.2235	739.2233	0.26	$C_{37}H_{38}O_{16}$		MS[2][739]:320.0675(100), 351.0857(60), 379.0803(26)	Procumbenoside M
86	14.33	397.1282	397.1282	0.08	$C_{22}H_{20}O_7$		MS[2][397]: 322.1194(80)	Cilinaphthalide A/Pronaphthalide A
87[#]	14.33	576.2080	576.2076	0.69	$C_{28}H_{30}O_{12}$		MS[2][576]:397.1281(100)	Cilinaphthalide A-glu
88	14.51	543.1499	543.1497	0.37	$C_{27}H_{26}O_{12}$		MS[2][543]:381.0965(100), 363.0859(43), 305.0805(36)	Justicidinoside C/Procumbenoside D/Cleistanthin B
89	14.62	675.1923	675.1920	0.44	$C_{32}H_{34}O_{16}$		MS[2][675]:381.0961(100), 363.0855(33), 333.0751(38), 305.0803(38)	Procumbenoside B or its isomer
90	14.88	411.1074	411.1074	-0.10	$C_{22}H_{18}O_8$		MS[2][411]:137.0231(100)	isomer of 6'-Hydroxy Justicidin A
91	14.88	590.1870	590.1868	0.24	$C_{28}H_{28}O_{13}$		MS[2][590]:137.0232(100), 411.1066(17)	Justicidinoside A
92	14.96	645.1816	645.1814	0.29	$C_{31}H_{32}O_{15}$		MS[2][645]:381.0965(100), 305.0801(57), 277.0857(39)	Procumbenoside A/Procumbenoside H
93	14.96	777.2236	777.2237	-0.03	$C_{36}H_{40}O_{19}$		MS[2][777]:381.0963(83), 305.0801(26), 277.0857(19)	Procumbenoside E /Ciliatoside A/Azizin
94	15.18	907.2528	907.2503	2.81	$C_{41}H_{48}O_{23}$	MS[2][907]:379.0824(100), 319.0612(33)		Ciliatoside B
95	15.35	395.0773	395.0761	2.94	$C_{21}H_{16}O_8$	MS[2][395]:351.0874(100), 322.0848(79), 308.0693(58), 336.06641(10)		9-Hydroxy-5-(4-hydroxy-3,5-dimethoxyphenyl)furo[3′,4′:6,7]naphtho[2,3-d][1,3]dioxol-6(8H)-one
96	15.36	645.1816	645.1814	0.29	$C_{31}H_{32}O_{15}$	MS[2][689]:379.0824(100), 319.0611(22), 290.0584(13)	MS[2][645]:381.0965(100), 363.0859(39), 305.0803(41), 277.0854(33)	Procumbenoside A/Procumbenoside H

97	15.53	411.1075	411.1074	0.15	$C_{22}H_{18}O_8$		MS^2[411]:137.0231(100)	isomer of 6'-Hydroxy Justicidin A
98	15.53	573.1606	573.1603	0.56	$C_{28}H_{28}O_{13}$		MS^2[573]:335.0909(100), 363.08558(88), 381.0963(79), 320.0672(46)	Justicidinoside A
99	15.74	327.2180	327.2166	4.28	$C_{18}H_{32}O_5$	MS^2[327]:211.1333(62), 229.1441(48), 171.1017(40), 85.0281(20)		Corchorifatty acid F
100#	15.89	919.2526	919.2503	2.55	$C_{42}H_{48}O_{23}$	MS^2[919]:379.0824(100)		Ciliatoside B + C
101	16.18	409.0931	409.0918	3.20	$C_{22}H_{18}O_8$	MS^2[409]:308.1055(100), 279.0663(80), 335.0926(66), 294.0899(62)		6'-Hydroxy Justicidin C
102	16.53	227.1286	227.1278	3.61	$C_{12}H_{20}O_4$	MS^2[227]:183.1382(100), 165.1276(17)		3Z-dodecenedioic acid
103	16.62	381.0968	381.0969	-0.10	$C_{21}H_{16}O_7$		MS^2[381]:307.0958(100), 337.1064(82), 279.1010(70)	Neojusticin C/Chinensinaphthol/4'-Demethylchinensinaphthol/Diphyllin/Isodiphyllin
104	16.89	329.2335	329.2323	3.80	$C_{18}H_{34}O_5$	MS^2[329]:211.1333(52), 229.1441(38), 171.1017(28)		Tianshic acid
105	17.08	397.1282	397.1282	0.00	$C_{22}H_{20}O_7$		MS^2[397]:348.0986(100),338.1143(67), 322.1194(62), 353.1375(43)	Cilinaphthalide A/Pronaphthalide A
106	17.77	411.1074	411.1074	-0.04	$C_{22}H_{18}O_8$		MS^2[411]:137.0231(100)	isomer of 6'-Hydroxy Justicidin A
107	17.85	411.1074	411.1074	-0.19	$C_{22}H_{18}O_8$		MS^2[411]:137.0231(100)	isomer of 6'-Hydroxy Justicidin A
108	18.05	397.1281	397.1282	-0.15	$C_{22}H_{20}O_7$		MS^2[397]:322.1194(59), 347.0908(49), 338.1142(47)	Cilinaphthalide A/Pronaphthalide A/
109	18.28	463.2327	463.2326	0.06	$C_{25}H_{34}O_8$		MS^2[463]:151.0752(100), 181.0856(47)	5-Methoxy-4,4'-di-O-methylsecolarciresinol
110	18.38	379.0825	379.0812	3.27	$C_{21}H_{16}O_7$	MS^2[379]:319.0611(100), 304.0377(80), 290.0585(41), 275.0352(34), 247.0398(33)	MS^2[381]:332.0670(100), 363.0856(92)	6'-Hydroxy Justicidin B
111	18.83	555.1498	555.1497	0.14	$C_{28}H_{26}O_{12}$	MS^2[555]:381.0964(22), 305.0804(15), 363.0858(7)		Diphyllin apioside-5-acetate
112	18.86	411.1071	411.1074	-0.83	$C_{22}H_{18}O_8$	MS^2[409]:308.1055(100),	MS^2[411]:335.0906(100),	6'-Hydroxy Justicidin A

						279.0663(73), 335.0926(64), 294.0899(53)	363.0855(89), 381.0961(66), 320.0672(61)	
113	19.36	381.0968	381.0969	-0.18	$C_{21}H_{16}O_7$		MS^2[381]:307.0957(100), 337.1064(83), 279.1010(69)	Neojusticin C/Chinensinaphthol/4'-Demethylchinensinaphthol/Diphyllin/Isodiphyllin
114	19.56	508.2543	508.2541	0.39	$C_{26}H_{34}O_9$		MS^2[508]:151.0753(100), 167.0700(60)	Justin C
115	19.77	363.0513	363.0499	3.80	$C_{20}H_{12}O_7$	MS^2[363]:289.0507(100),350.0432(58), 317.0455(46), 261.0557(42)		Taiwanin E
116	19.90	411.1437	411.1438	-0.29	$C_{23}H_{22}O_7$		MS^2[411]:336.1351(75), 362.1148(75), 321.1122(48)	Cilinaphthalide B
117*	20.04	365.1007	365.1020	-3.59	$C_{21}H_{16}O_6$		MS^2[365]:321.1117(75), 275.0699(43), 303.0648(45), 335.0911(33)	Justicidin B
118	21.02	395.1123	395.1125	-0.56	$C_{22}H_{18}O_7$		MS^2[395]:320.1040(69), 346.0829(62), 336.0985(62), 380.0882(30)	Justicidin C/Phyllamyricin C/Chinensinaphthol methyl ether/5'-Methoxy retrochinensin/Procumphthalide A
119	21.02	492.2226	492.2228	-0.49	$C_{25}H_{30}O_9$		MS^2[492]:135.0439(100), 167.0700(79)	Justin B
120	21.16	570.2598	570.2599	-0.16	$C_{33}H_{35}N_3O_6$		MS^2[570]:131.0490(100),120.0808(82)	Justicianene A
121*	21.27	395.1123	395.1125	-0.56	$C_{22}H_{18}O_7$		MS^2[395]: 320.1040(69), 336.0985(62), 305.0804 (33), 380.0883(30)	Justicidin A
122	21.38	395.1124	395.1125	-0.30	$C_{22}H_{18}O_7$		MS^2[395]:320.1040(69), 346.0829(62), 336.0985(62), 380.0882(30)	Justicidin C/Phyllamyricin C/Chinensinaphthol methyl ether/5'-Methoxy retrochinensin/Procumphthalide A
123	21.66	522.2695	522.2698	-0.42	$C_{27}H_{36}O_9$		MS^2[522]:151.0752(100), 181.0856(77)	5-Methoxy-4,4'-di-O-methylsecolarciresinol diacetate
124	21.66	475.2324	475.2326	-0.46	$C_{26}H_{34}O_8$		MS^2[475]:151.0752(100)	5-Methoxy-4,4'-di-O-methylsecolarciresinol diacetate

125	21.75	492.2589	492.2592	-0.59	$C_{26}H_{34}O_8$		MS^2[492]:151.0752(69), 217.1222(7)	Secoisolariciresinol dimethyl ether diacetate
126	21.92	395.1124	395.1125	-0.30	$C_{22}H_{18}O_7$		MS^2[395]:319.0961(100), 347.0909(54), 332.0670(38)	Justicidin C/Phyllamyricin C/Chinensinaphthol methyl ether/5'-Methoxy retrochinensin/Procumphthalide A
127	22.12	379.0809	379.0812	-0.84	$C_{21}H_{14}O_7$		MS^2[379]:305.0802(44), 335.0910(28)	Justicidin D/Taiwanin C/Taiwanin E methyl ether
128	22.52	489.2116	489.2119	-0.61	$C_{26}H_{32}O_9$		MS^2[489]:135.0439(100), 181.0857(62)	(-)-Dihydroclusin diacetate
129	22.63	379.0811	379.0812	-0.32	$C_{21}H_{14}O_7$		MS^2[379]:305.0802(44), 335.0910(28)	Justicidin D/Taiwanin C/Taiwanin E methyl ether
130	23.15	293.2127	293.2111	5.53	$C_{18}H_{30}O_3$	MS^2[293]:275.2016(47),195.1383(22), 235.1700(19),223.1337(15)		13-HOTrE or its isomer
131	23.20	293.2127	293.2111	5.22	$C_{18}H_{30}O_3$	MS^2[293]:275.2016(47),195.1383(22), 235.1700(19),223.1337(15)		13-HOTrE or its isomer
132	23.96	295.2281	295.2268	4.47	$C_{18}H_{32}O_3$	MS^2[295]:277.2172(57),195.1383(34), 171.1017(22)		13-HODE or its isomer

* Identified by comparing with reference standards; # Potential new compounds

Table S2. Detailed information of the 10 reference standards

Compound Name	Formula	Lot Number	Supplier
Neochlorogenic acid	$C_{16}H_{18}O_9$	MUST-15011412	Chengdu Master Biotechnology Co., Ltd
Chlorogenic acid	$C_{16}H_{18}O_9$	L-007-171216	Chengdu Ruifensi Biotechnology Co., Ltd
Azelaic acid	$C_9H_{16}O_4$	R-064	Chengdu Herpurify Co.,Ltd
Rutin	$C_{27}H_{30}O_{16}$	AF8032520	Chengdu Efa Biotechnology Co., Ltd
Hyperoside	$C_{21}H_{20}O_{12}$	111521-201507	National Institutes for Food and Drug Control
Isoquercitrin	$C_{21}H_{20}O_{12}$	Y-076-181025	Chengdu Herpurify Co.,Ltd
Orientin	$C_{21}H_{20}O_{11}$	H-044-181025	Chengdu Herpurify Co.,Ltd
Baicalin	$C_{21}H_{18}O_{11}$	DST190312-024	Chengdu desite biological technology Co., Ltd
Justicidin B	$C_{21}H_{16}O_6$	CFN95701	Wuhan ChemFaces Biotechnology Co., Ltd
Justicidin A	$C_{22}H_{18}O_7$	CFN95702	Wuhan ChemFaces Biotechnology Co., Ltd
